# The cardioprotective effect of *S. africana caerulea*/Blue Sage in ischaemia and reperfusion induced oxidative stress

**DOI:** 10.3389/fphar.2023.1254561

**Published:** 2023-09-25

**Authors:** Ruduwaan Salie, John Lopes, Leon Kotze, Ruzayda van Aarde

**Affiliations:** ^1^ South African Medical Research Council, Biomedical Research and Innovation Platform, Cape Town, Western Cape, South Africa; ^2^ Faculty of Medicine and Health Sciences, Stellenbosch University, Cape Town, Western Cape, South Africa

**Keywords:** myocardial ischaemia, reperfusion injury, cardioprotection, Blue Sage (BLS), traditional medicine

## Abstract

**Background:** Since antiquity, alternative herbal remedies, such as *S. africana caerulea*/Blue Sage (BLS) water infusion extract (WIE) has been used by traditional healers, for the effective treatment of various chronic inflammatory disorders associated with reduced cellular antioxidant defense mechanisms and free radical cellular damage. In the heart, ischaemia—reperfusion (I/R) induced oxidative stress becomes an early crucial event in the pathogenesis of ischaemia—reperfusion injury (I/RI) and subsequent heart failure.

**Purpose/Aim:** To investigate whether BLS WIE treatment during ischaemia and/or reperfusion may be cardioprotective.

**Study design:** Isolated perfused rat hearts were exposed to 35 min regional ischaemia (RI) and 60 min reperfusion. The BLS WIE was applied: i) for the last 10 min of RI (PerT) or ii) from onset of reperfusion (PostT) or iii) both (PerT) + (PostT). Methods: Endpoints were functional recovery and infarct size (IS). In another set of experiments, left ventricles were freeze-clamped after RI and 10 min reperfusion for detection of total and phosphorylated p-ERK p44/p42, p-Akt, p-p38-MAPK, p-JNK, Nrf-2, NF-kB, Bax, Bcl-2, Caspase-3, and PGC-1α by Western blot analysis.

**Results:** BLS (PostT) significantly increased ERK p44, p-Akt, Nrf-2, and Bcl-2 levels; significantly decreased p-p38-MAPK as well as p-JNK p46 phosphorylation; did not affect Bax levels and significantly decreased Bax/Bcl-2 ratios. This was associated with significantly reduced Caspase-3 levels and increased PGC-1α phosphorylation, particlarly when BLS WIE was administered as PostT.

**Conclusion:** The administration of polyphenol-rich BLS WIE at different stages of ischaemia and/or reperfusion, activate/inhibit several signaling events simultaneously and mediate cardioprotection in a multitarget manner.

## Introduction

In the South African context, the self-care system of any chronic disease, including diabetes, can be physically, emotionally and especially financially very difficult. Even though these illnesses can be treated and managed clinically, first-line treatments are often associated with numerous side effects. Consequently, the application of alternative herbal remedies, has been reported to be effective and simple for the treatment of many chronic ailments (Arendse, 2013; Department of Biodiversity, 2013).

The use of traditional medicine/alternative herbal remedies was endorsed by the World Health Organization (WHO) Traditional Medicine Strategy (2005) with future possibilities of plant extracts as substitute therapy. It is common knowledge since antiquity that the application of alternative herbal remedies, has been simple effective treatments of various chronic ailments. One such alternative herbal remedy, is *S. africana caerulea*/Blue Sage (BLS). There is no lack in the validation of its use but there is a lack of scientific information regarding the pharmacological/physiological properties of BLS for example, to corroborate its traditional use and to extend the use of this plant into new therapeutic applications. This necessitates further research into BLS, which exhibits promising *in vitro* activity in various research models (Kamatoua et al., 2008). BLS has significant antioxidant capacity that has the potential to effectively ameliorate inflammatory related disorders such as diabetes mellitus, neurodegenerative diseases, cancer and heart disease, all associated with reduced cellular antioxidant defense mechanisms and ensuing free radical cellular damage (Hess and Manson, 1984).

Low levels of reactive oxygen species (ROS) are constantly being generated in cardiac myocytes and serve as vital intracellular regulators, whereas excessive ROS is neutralized by endogenous antioxidant mechanisms. However, I/R can induce alterations in the redox balance and oxidative stress becomes an early crucial event in the pathogenesis of several cardiac disorders, I/RI and subsequent heart failure. Notably, it is not always possible to completely counteract the damaging effects of oxidative stress, mainly due to the many sources of ROS. However, it may be possible to regulate ROS signaling pathways in such a manner to allow essential cell function and simultaneously prevent oxidative stress induced I/R cardiac damage. This may be achieved by means of a multitarget cardioprotective approach using BLS WIE, which contains several polyphenolic compounds applied at different stages of I/R. In this manner several signaling cascades can be simultaneously targeted to ensure an improved cardioprotective effect. Consequently, this study investigated the antioxidant capacity of BLS in ischaemia and reperfusion induced oxidative stress in the *ex vivo* working Wistar rat heart model.

## Materials and methods

### Animal ethics and regulations

Animal ethics was obtained from the Ethics Committee for Research on Animals (ECRA): ref 13–18, South African Medical Research Council (SAMRC). Experimental animals were used in accordance to ethical guidelines as set out by the ECRA, SAMRC Guideline Book 3: 1990—Use of Animals in Research and the South African National Standard for Care and Use of Animals for Scientific Purpose (SANS 10386: 2008). The rats had free access to food and water before experimentation. Rats were anaesthetised with sodium pentobarbital (120 mg/kg) by intraperitoneal injection before surgical removal of the hearts.

Preparation of BLS WIE: 1 L of boiling water was added onto 200 g of BLS plant material and extracted for 3 days at room temperature. The plant material was separated from the BLS WIE, which was subjected to Liquid chromatography–mass spectrometry (LC-MS) analysis (Figures 2A–C).

**FIGURE 1:**
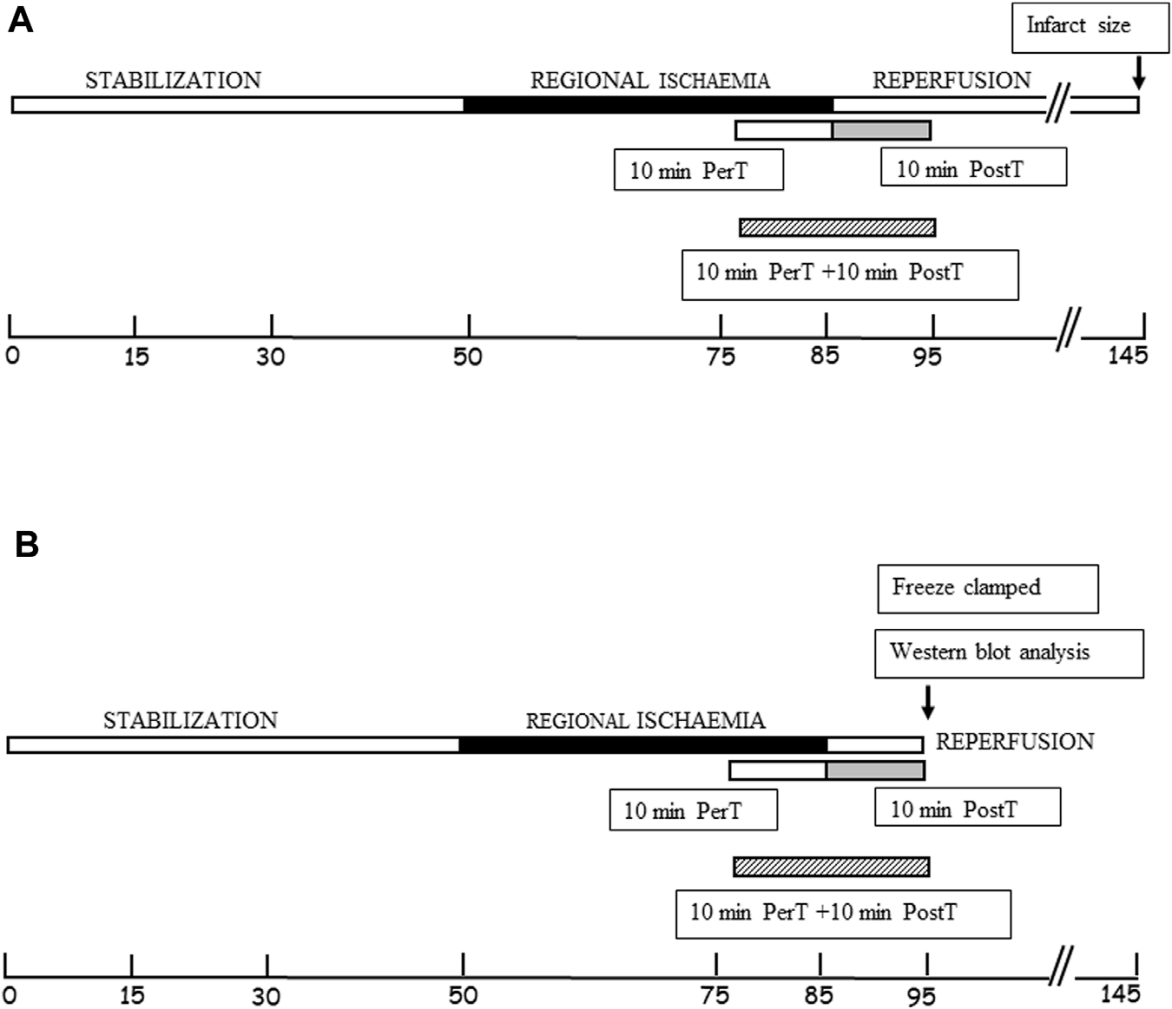
**(A)** Schematic representation of BLS WIE administration to the PerT, PostT, and PerT + PostT groups, followed by infarct size and functional recovery measurements as endpoints. **(B)** Schematic representation of BLS WIE PerT, PostT, and PerT + PostT, showing time points of freeze clamping for Western blot analysis.

**FIGURE 2:**
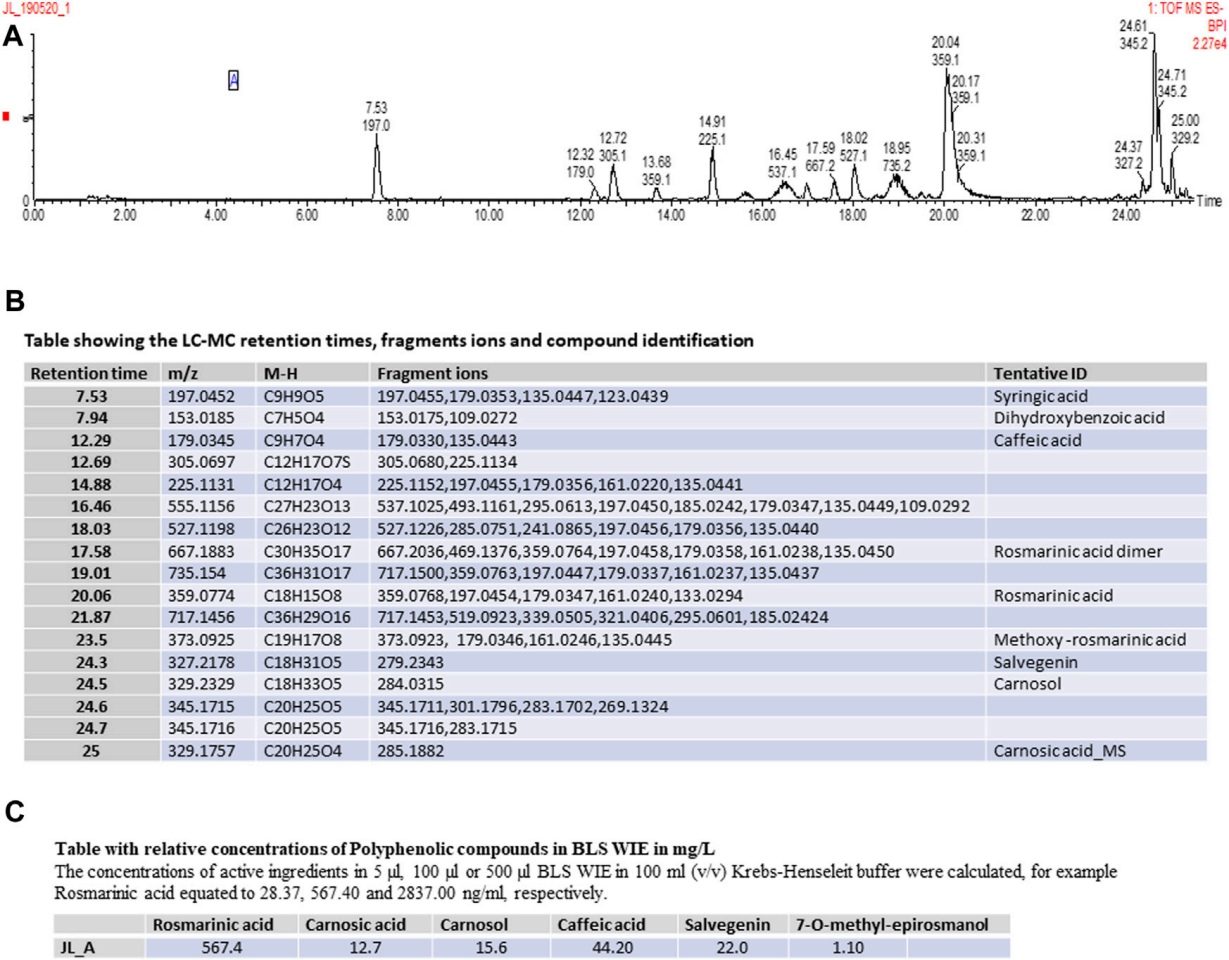
**(A)** Schematic representation of LC-MS Spectrum, **(B)** Table showing the LC-MC retention times, fragments ions and compound identification, **(C)** Table showing the relative concentrations Polyphenolic compounds in BLS WIE in mg/L.

Experimental design: BLS WIE dose response (*n* = 6 per dosage) Three different concentrations of BLS WIE (5 μL, 100 µL or 500 µL in 100 mL (v/v) Krebs-Henseleit buffer) were tested by administration for 10 min at the onset of reperfusion, as a post-treatment (PostT) after RI to determine the most appropriate cardioprotective dosage using Infarct size (IS) and Functional recovery (FR) as end-points (Figure 1A). The concentrations of active ingredients (Figures 2A–C) in 5 μL, 100 µL or 500 µL BLS WIE in 100 mL (v/v) Krebs-Henseleit buffer were calculated, for example Rosmarinic acid equated to 28.37, 567.40, and 2837.00 ng/mL, respectively.

### Experimental groups for RI

#### Untreated (UT) group (*n* = 6)

In the untreated group (UT), isolated rat hearts were stabilized for 45 min, followed by 35 min RI and 60 min reperfusion.

#### Per-treatment group (PerT) (*n* = 6)

Per-treated (PerT) hearts were exposed to a 45 min stabilization period, 35 min RI, subjected to a BLS WIE treatment during the last 10 min of RI and followed by 60 min reperfusion.

#### Post-treatment group (PostT) (*n* = 6)

Post-treated (PostT) hearts were exposed to a 45 min stabilization period, 35 min RI and BLS WIE treatment for 10 min at the onset of the 60 min reperfusion after RI.

#### Per- and post-treatment group (PerT + PostT) (*n* = 6)

Per- and post-treatment (PerT + PostT) hearts were exposed to a 45 min stabilization period (Arendse, 2013), 35 min RI and subjected to a BLS WIE treatment during the last 10 min of RI as well as for 10 min at the onset of the 60 min reperfusion after RI.

### Perfusion technique and IS determination

Hearts were perfused as previously described by Lochner et al., 1999. The percentage functional recovery (% FR) of hearts was determined by expressing the post-ischaemic cardiac output (CO), calculated as Qc + Qa rates in mL/min, as a percentage of the pre-ischaemic CO.

**FIGURE 3:**
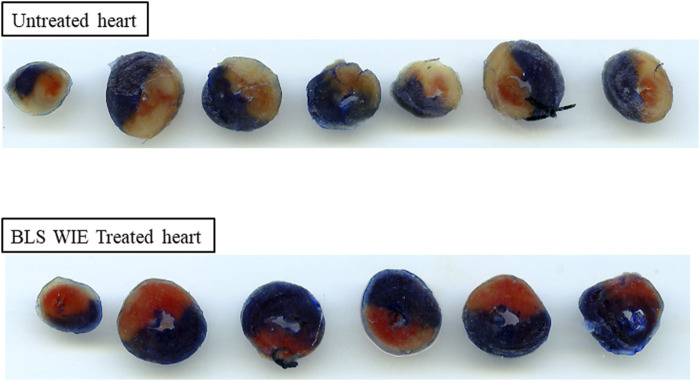
Representative images of heart slices for untreated and BLS WIE treated hearts. The infarcted area, calculated as % IS outlined as unstained area, area at risk (red color) and the viable area (dark blue). The BLS WIE treated heart shows significantly reduced % IS when compared to untreated heart, illustrating the cardioprotective effects of the BLS WIE.

At the completion of regional ischaemia and reperfusion, the silk suture around the LAD was permanently tied and a 0.25% Evan’s blue solution infused into the heart to outline viable tissues. Hearts were removed, frozen, cut into 2 mm thick transverse tissue segments and incubated in 1% triphenyl tetrazolium chloride (TTC) in phosphate buffer, pH 7.4 for 10 min. Damaged tissues take on a deep red coloration. Infarcted tissue areas are not stained and have a white color. The reaction with TTC was stopped by placing the tissue segments in 10% formalin. Tissue segments were placed between two glass plates and ImageJ software was used to outline and analyze the left ventricle area at risk (AR) (red colored area), area of infarct tissue (I) (unstained white area) and area of viable tissue (blue outlined area). The infarct size (IS) was determined and expressed as a percentage of the area at risk (% I/AR) (Figure 3).

### Experimental groups for western blot analysis

After each BLS WIE treatment regiments (PerT, PostT, and PerT + PostT) and RI, the left ventricles were freeze-clamped at 10 min of reperfusion (Figure 1B). Freeze-clamped left ventricular tissue was used for subsequent detection and measurement of total and phosphorylated ERK p44/p42, p-Akt, p38-MAPK, JNK, Nrf-2, NF-kB, Bax, Bcl-2, Caspase-3, and PGC-1α by Western blot analysis (Gel electrophoresis). Samples were subjected to electrophoresis on a 12% or 7.5% polyacrylamide gel (SDS–PAGE), depending on the size of the protein of interest, using the standard BIO-RAD Mini Protean III system. The separated proteins were transferred to an Immobilon membrane (Millipore) (Billerica, MA, United States: Polyvinylidenedifluoride (PVDF) membrane), using the Trans-Blot®Turbo™ Transfer system. Non-specific binding sites on the membrane were blocked with 5% fat free milk (5 g) in TBS-Tween (Tris-buffered saline +0.1% Tween 20) for 1-2 h at room temperature and incubated overnight at 4°C with the primary antibodies (Cell Signaling Technology, Massachusetts, United States) that recognize total or phosphorylated proteins: ERK p44/p42, Akt, p38-MAPK, JNK, Nrf-2, NF-κB, Bax, Bcl-2, Caspase-3, and PGC-1α. The membranes were washed with TBS-T (3 × 5 min) and then incubated at room temperature with a diluted horseradish peroxidase-labelled secondary antibody (Cell Signaling Technology). After thorough washing with TBS-T, membranes were covered with Enhanced chemiluminescence ECL detective reagent for 1 minute and briefly exposed with the Chemidoc MP Imager System with Image lab 5. Stain-Free membranes and the Chemidoc MP Imager System with Image lab 5 were used to validate protein transfer and equal loading of samples. Note that, densitometry measurements were normalized to those of beta tubulin and the fold change is presented as a comparison to the Untreated group.

#### Negative control group (-ve C) (*n* = 6)

In the negative control group (-ve C), isolated rat hearts were stabilized for 45 min and left ventricles freeze-clamped.

#### Untreated group (UT) (*n* = 6)

In the untreated group (UT), isolated rat hearts were stabilized for 45 min, followed by 35 min RI, after which the left ventricles were freeze-clamped at 10 min of reperfusion.

#### Per-treatment group (PerT) (*n* = 6)

BLS WIE was administered during the last 10 min of RI and freeze-clamped at 10 min of reperfusion.

#### Post-treatment group (PostT) (*n* = 6)

BLS WIE was administered for 10 min at the onset of reperfusion after RI and freeze-clamped at the end of this period.

### Per- and post-treatment group (PerT + PostT) (*n* = 6)

BLS WIE was administered during the last 10 min of RI as well as for 10 min at the start of reperfusion after RI and freeze-clamped at the end of this period.

### Statistical analysis

Results were expressed as mean ± standard error of the mean (SEM). For multiple comparison one-way analysis of variance (ANOVA) was utilised (Graph Pad Prism Plus Version 8.0.1 software). Post-hoc testing for differences between selected groups was done using Bonferroni’s method. A *p*-value of <0.05 was considered significant.

## Results

LC-MS analysis of BLS WIE extract identified the following Polyphenolic compounds (Figures 2A–C).

### The area at risk zone

The AAR represents the entire myocardial perfusion bed distal to an occluded coronary artery and is a major determinant of final IS and prognosis (Califf et al., 1985). However, the variability of both the branching pattern and myocardial vascular regions supplied by the LAD makes consistent reproducibility in the size of the AAR difficult, which necessitates consistent positioning of the ligature around the LAD (Kanno et al., 2002). Subsequently, a similar AAR among experimental groups represents a consistent positioning of the ligature around the LAD and in this study the area at risk zone (49.85% ± 1.29%), expressed as a percentage of the left ventricular area was similar in untreated and all BLS WIE treated groups, implying that results are comparable.

### The effect of BLS WIE on % infarct size

**FIGURE 4:**
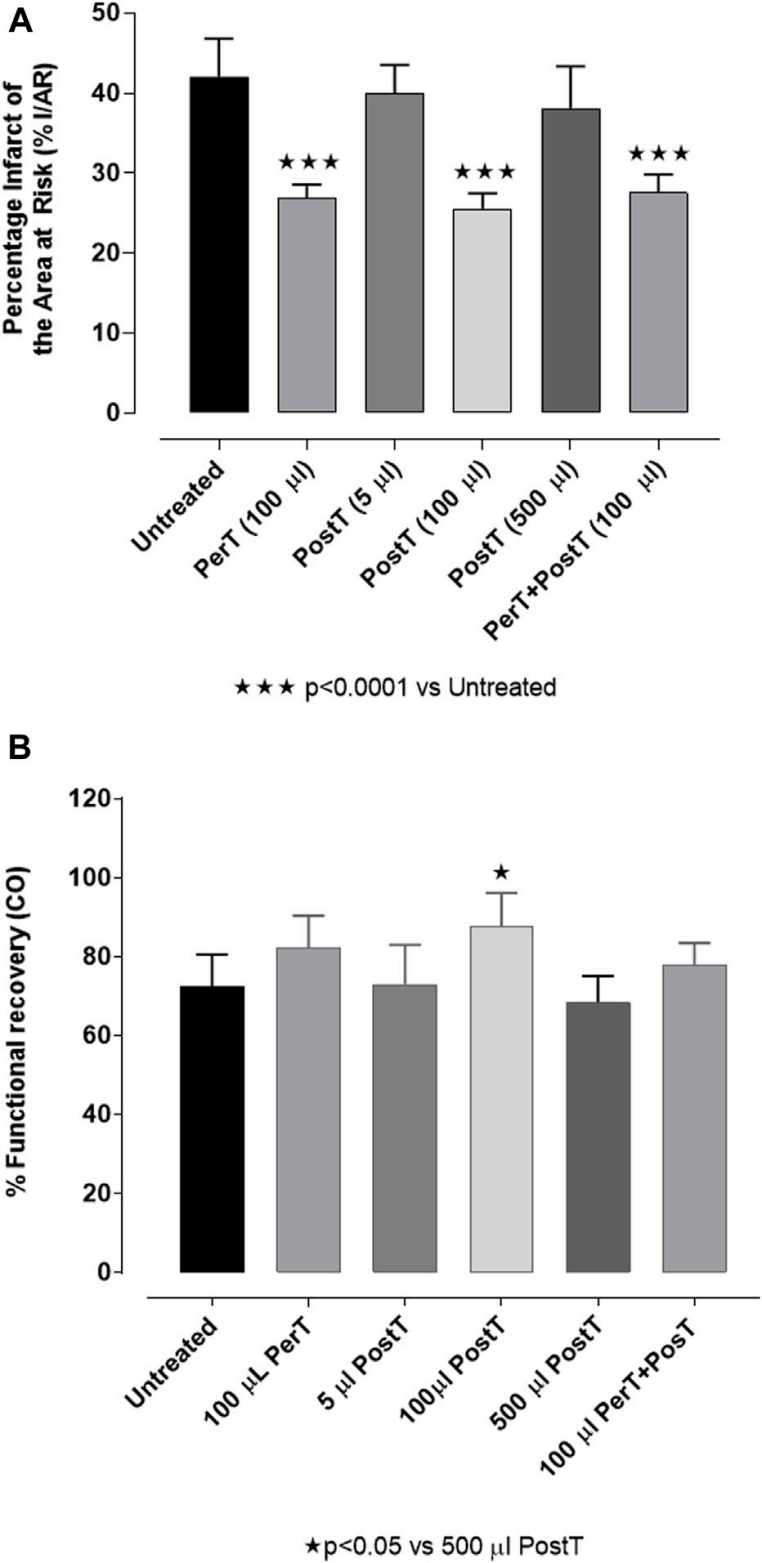
**(A)** BLS WIE dose response on % IS: BLS post-treatment (PostT): 5 μl, 100 μl or 500 μl BLS WIE in 100 ml Krebs-Henseleit buffer. There is an infarct sparing effect with 100 μl BLS WIE when applied as PerT, PostT, and PerT + PostT. **(B)** BLS WIE dose response on % FR (CO): BLS post-treatment (PostT): 5 μl, 100 μl or 500 μl BLS WIE in 100 ml in Krebs-Henseleit buffer; Functional recovery of 100 μl BLS WIE when applied as PerT, PostT, and PerT + PostT.

BLS WIE was initially applied at the onset of reperfusion, as a PostT after RI at 5 μL, 100 µL or 500 µL in 100 mL (v/v) Krebs-Henseleit buffer to identify a protective dose. Hearts treated with 100 µL BLS PostT significantly reduced % IS (25.57% ± 0.77%) when compared to untreated hearts (42.07% ± 1.92%, *p* < 0.0001), while hearts treated with 5 µL (39.99 ± 1.57) or 500 µL (38.09% ± 2.34%) BLS had no effect on IS and were similar to untreated hearts (42.07% ± 1.92%), (Figure 4A). Due to the protective effect elicited with 100 μL BLS, all subsequent experiments were performed with 100 µL BLS. The application of 100 µL BLS during the last 10 min of RI (BLS PerT: 26.86% ± 0.70%) or for 10 min at the onset of reperfusion after RI (BLS PostT: 25.57% ± 0.77%) or during the last 10 min of RI and continued during the first 10 min of reperfusion (BLS PerT + PostT: 27.55 ± 0.92), resulted in a significant reduction in IS when compared to the untreated group (42.07% ± 1.92%, *p* < 0.0001), (Figure 4A).

### The effect of BLS WIE on % FR (CO)

BLS WIE: BLS administered at 5 µL or 500 µL for 10 min at the onset of reperfusion, as a PostT after RI, had no effect on % FR (72.92% ± 4.43% and 67.67% ± 3.14%, respectively) when compared to untreated hearts (72.51% ± 3.23%), (Figure 4B). However, 100 µL BLS PostT, significantly increased % FR when compared to the group of hearts treated with 500 µL BLS WIE (PostT) (83.49% ± 2.98% vs. 67.50% ± 3.23%, *p* < 0.05), (Figure 4B).

### p-ERK p-44/p42, p-Akt, p-Bax, p-Bcl, p-p38-MAPK, p-JNK, p-Nrf-2, p-NF-kB, p-Caspase-3, and p-PGC-1α activation

#### p-ERK p44/p42

**FIGURE 5:**
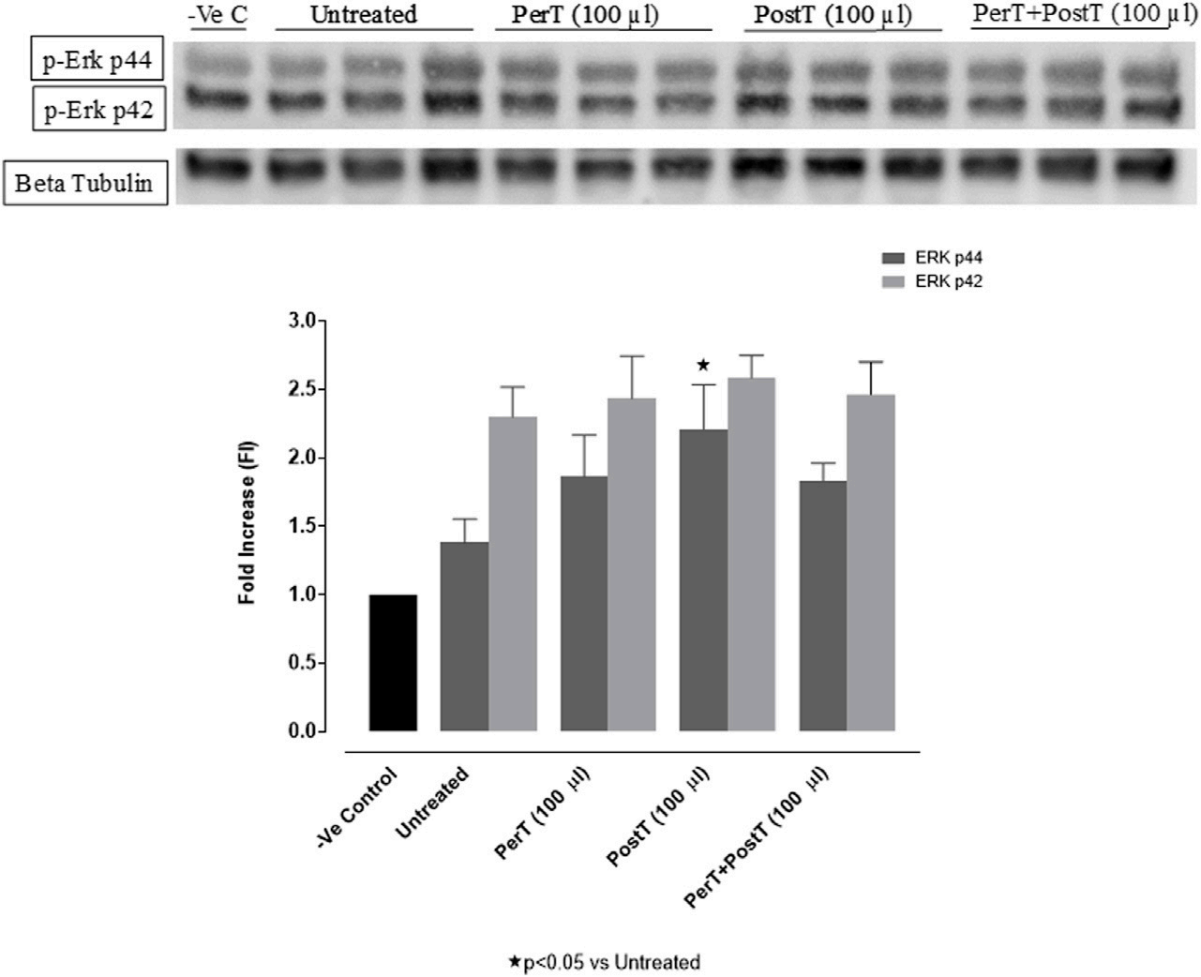
p-ERK p44/p42 activation at 10 min of reperfusion for experimental groups: Negative control (-Ve C), Untreated, BLS PerT, BLS PostT, and BLS PerT + PostT.

BLS PostT (FI: 2.21 ± 0.18) significantly increased p-ERK p44 phosphorylation when compared to the untreated group (FI: 1.36 ± 0.10, *p* < 0.05). BLS PerT (FI: 1.86 ± 0.17) or BLS PerT + PostT (FI: 1.83 ± 0.07) had no effect on p-ERK p44 phosphorylation. Although, BLS PerT, BLS PostT or BLS PerT + PostT had no effect on p-ERK p42 activation when compared to the untreated group, it was found to be significantly activated relative to the -Ve control (Figure 5).

#### p-Akt

**FIGURE 6:**
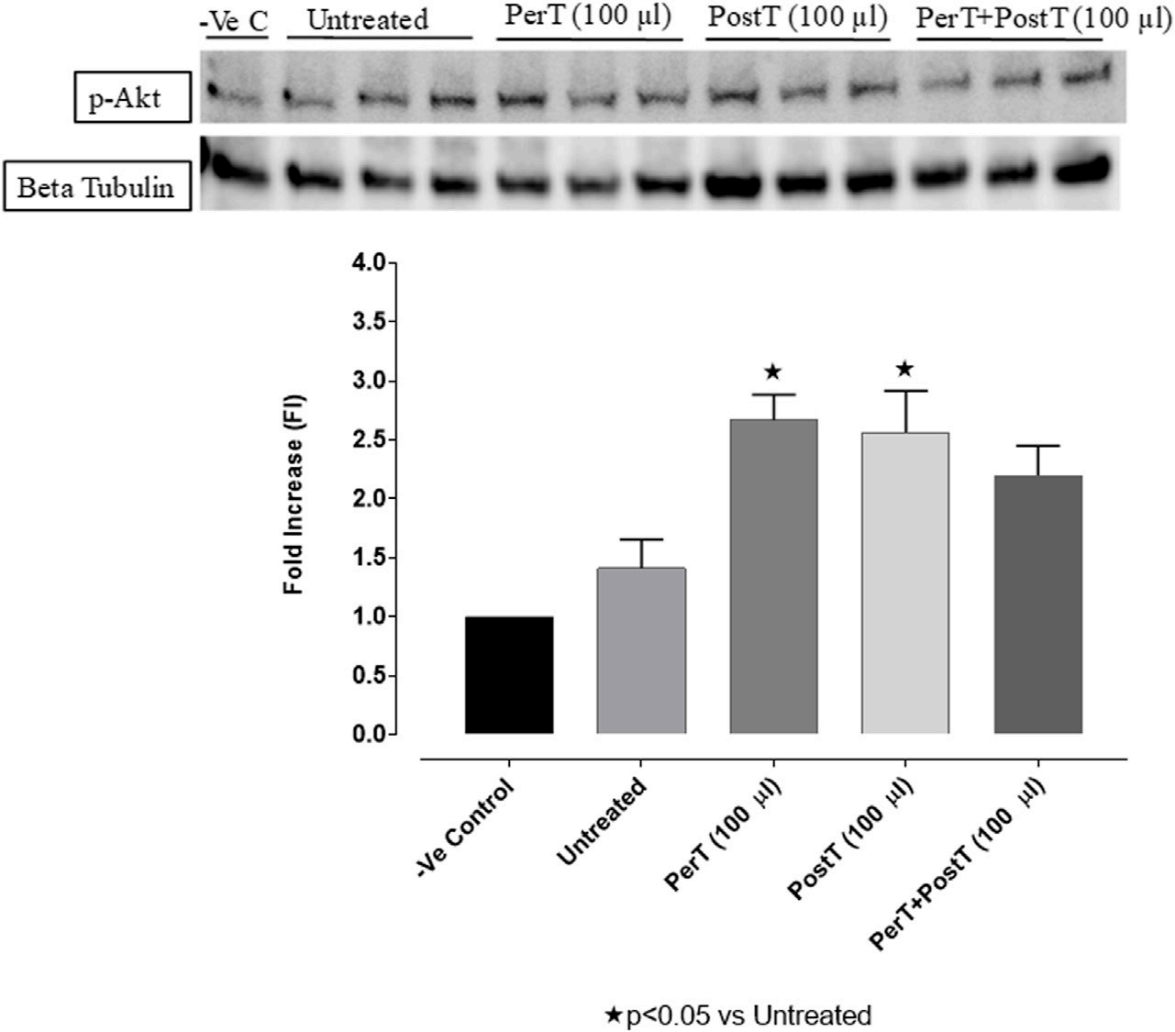
p-Akt activation at 10 min of reperfusion for experimental groups: Negative control (-Ve C), Untreated, BLS PerT, BLS PostT, and BLS PerT + PostT.

The administration of BLS as a PerT (FI: 2.66 ± 0.25) and PostT (FI: 2.83 ± 0.30) significantly increased p-Akt phosphorylation when compared to the untreated group (FI: 1.73 ± 0.27, *p* < 0.05). Although BLS PerT + PostT (FI: 2.71 ± 0.15) had no effect on p-Akt activation, it was not significantly reduced when compared to BLS PerT or PostT (Figure 6).

#### p-p38-MAPK

**FIGURE 7:**
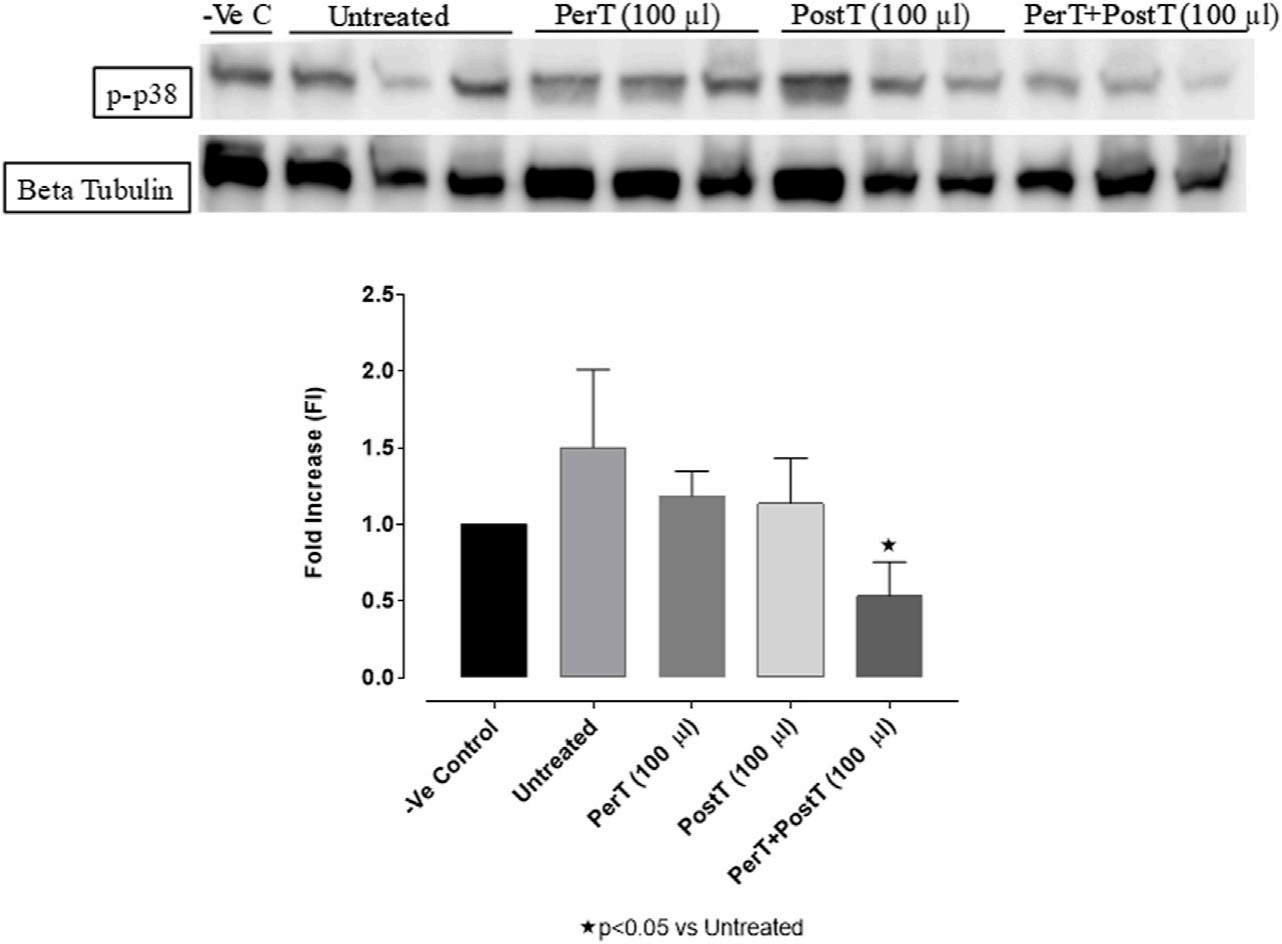
p-p38-MAPK activation at 10 min of reperfusion for experimental groups: Negative control (-Ve C), Untreated, BLS PerT, BLS PostT, and BLS PerT + PostT.

BLS PerT (FI: 1.27 ± 0.09) and BLS PostT (FI: 1.14 ± 0.16) had no effect on p38-MAPK phosphorylation. However, p-p38-MAPK phosphorylation was significantly decreased when BLS was applied as a PerT + PostT (FI: 0.53 ± 0.12) treatment (Untreated group FI: 1.50 ± 0.03, *p* < 0.05) (Figure 7).

#### p-JNK

**FIGURE 8:**
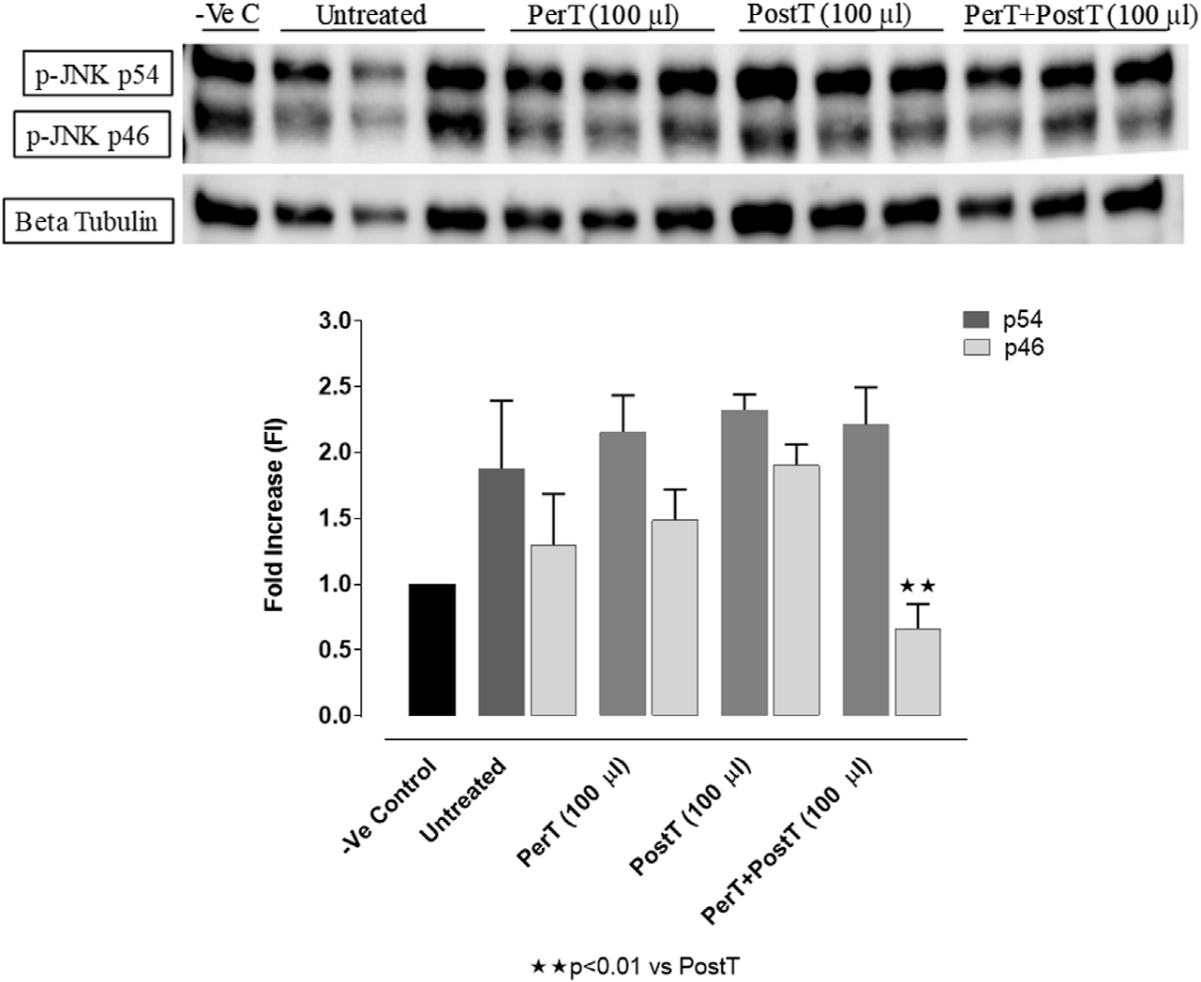
p-JNK activation at 10 min of reperfusion for experimental groups: Negative control (-Ve C), Untreated, BLS PerT, BLS PostT, and BLS PerT + PostT.

BLS WIE as a PerT, PostT and PerT + PostT had no effect on the phosphorylation of p-JNK p54. Similarly, p-JNK p46 activation was not affected by BLS PerT or PostT. However, BLS PerT + PostT significantly decreased p-JNK p46 activation compared to the untreated group (FI: 0.66 ± 0.16 vs. 1.17 ± 0.22, *p* < 0.01) (Figure 8).

#### Bax

**FIGURE 9:**
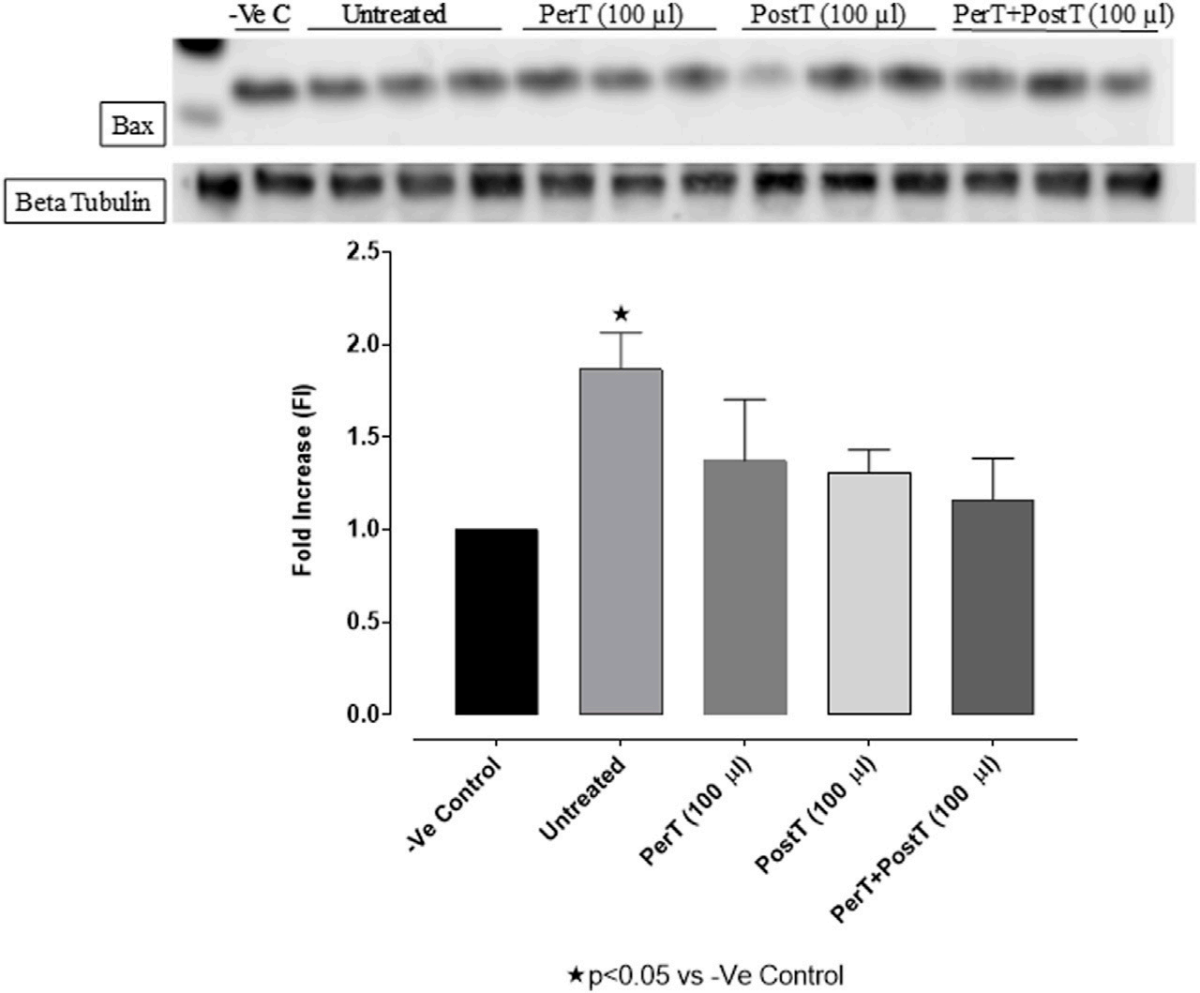
Bax activation at 10 min of reperfusion for experimental groups: Negative control (-Ve C), Untreated, BLS PerT, BLS PostT, and BLS PerT + PostT.

Bax was significantly activated in the untreated group (FI: 1.47 ± 0.04), whereas BLS PerT (FI: 1.11 ± 0.16), BLS PostT (FI: 0.98 ± 0.19) or BLS PerT + PostT (FI: 1.17 ± 0.17) marginally but not significantly decreased Bax activation when compared to the untreated group (Figure 9).

#### Bcl-2

**FIGURE 10:**
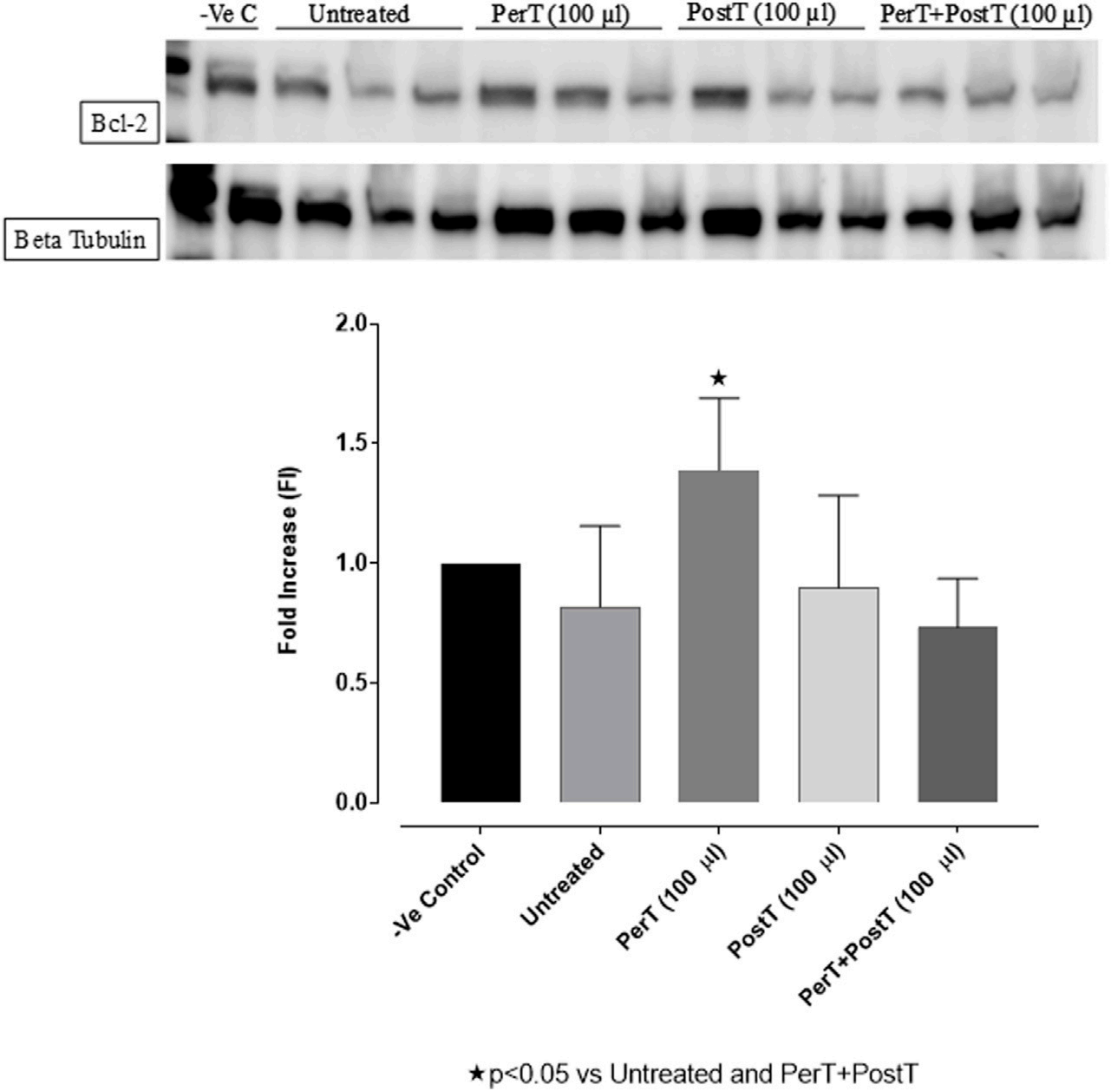
Bcl-2 activation at 10 min of reperfusion for experimental groups: Negative control (-Ve C), Untreated, BLS (PerT), BLS (PostT), and BLS (PerT + PostT).

BLS as a PerT (FI: 1.39 ± 0.17) significantly activated Bcl-2 vs. Untreated (FI: 0.82 ± 0.19, *p* < 0.05) (Figure 10).

#### Bax/Bcl-2 ratio

**FIGURE 11:**
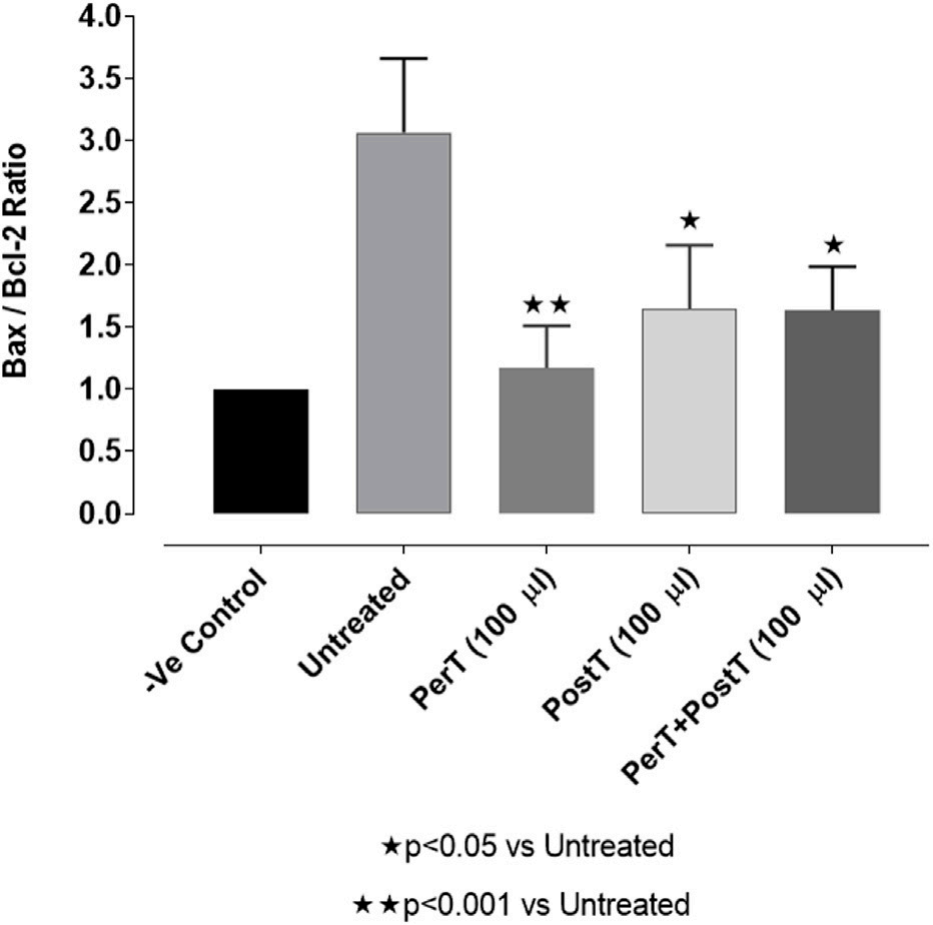
Ratio of pro- and anti-apoptotic factors Bax/Bcl-2 at 10 min reperfusion for the for experimental groups: Negative control (-Ve C), Untreated, BLS (PerT), BLS (PostT), and BLS (PerT + PostT).

Comparing the relative Bax to the Bcl-2 expression level (Bax/Bcl-2 ratio), it was found that the Bax/Bcl-2 ratio was significantly decreased with BLS (PerT), whereas the Bax/Bcl-2 ratio of BLS PostT as well as BLS (PerT + PostT) showed marginal but not significant decreases (Figure 11).

#### NF-kB

**FIGURE 12:**
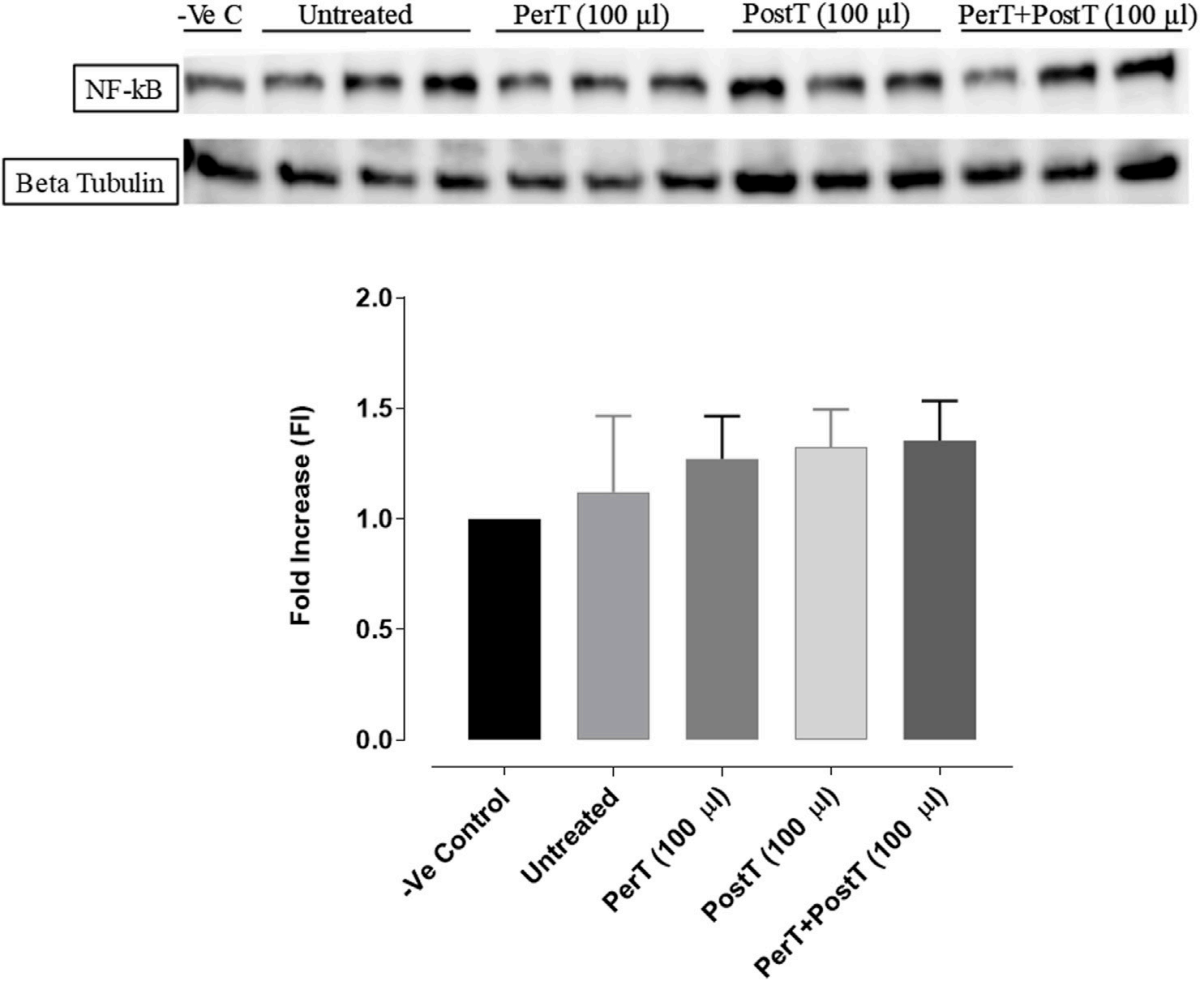
NF-kB activation at 10 min of reperfusion for experimental groups: Negative control (-Ve C), Untreated, BLS (PerT), BLS (PostT), and BLS (PerT + PostT).

BLS PerT, BLS PostT or BLS PerT + PostT had no effect on NF-kB phosphorylation when compared to the untreated group (Figure 12).

#### Nrf-2

**FIGURE 13:**
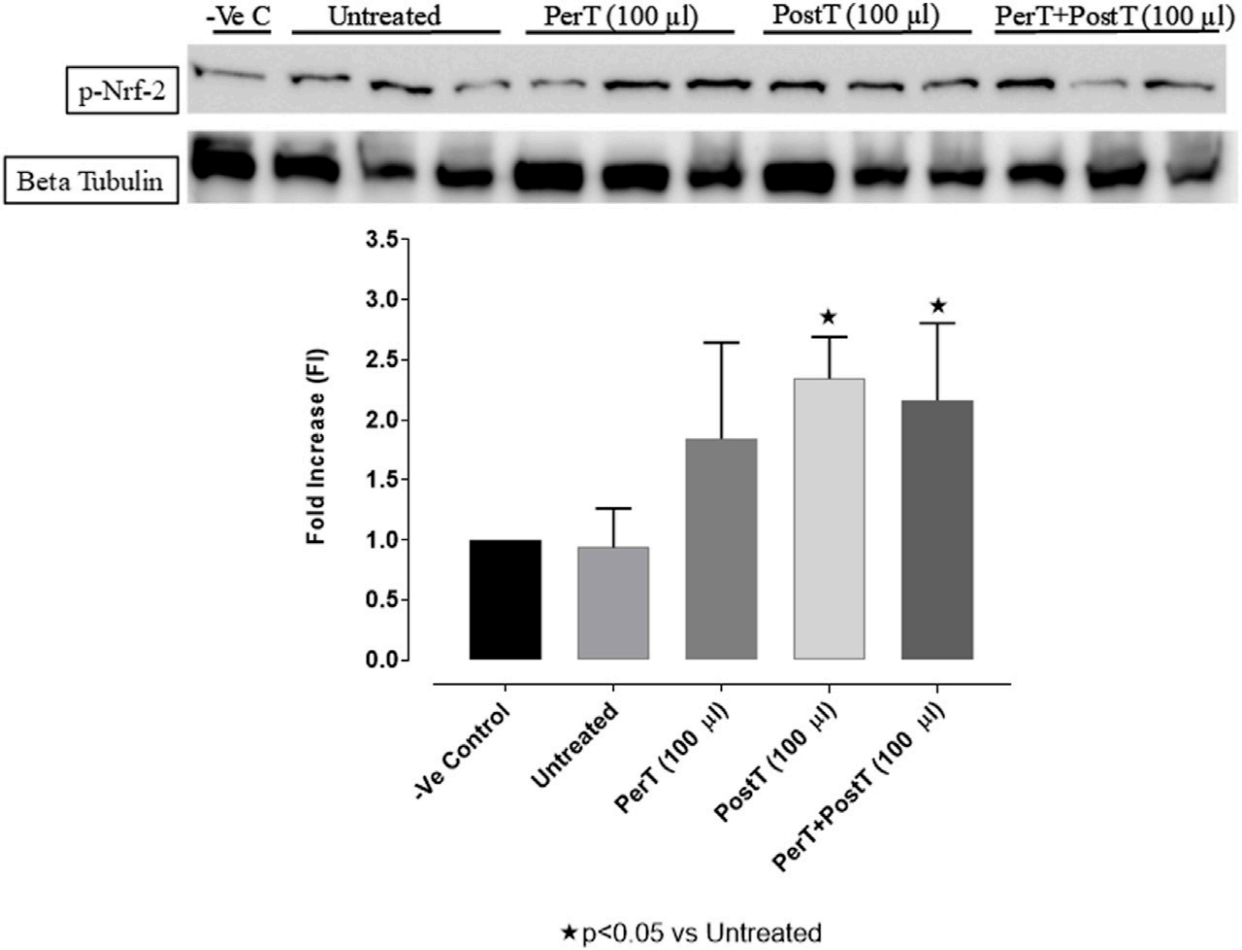
Nrf-2 activation at 10 min of reperfusion for experimental groups: Negative control (-Ve C), Untreated, BLS per-treatment (PerT), post-treatment (PostT), and BLS per- and post-treatment (PerT + PostT).

BLS PostT (FI: 2.34 ± 0.19) as well as BLS PerT + PostT (FI: 2.19 ± 0.34) increased Nrf-2 phosphorylation significantly when compared to the untreated group (FI: 0.93 ± 0.18, *p* < 0.05) (Figure 13).

#### Caspase-3

**FIGURE 14:**
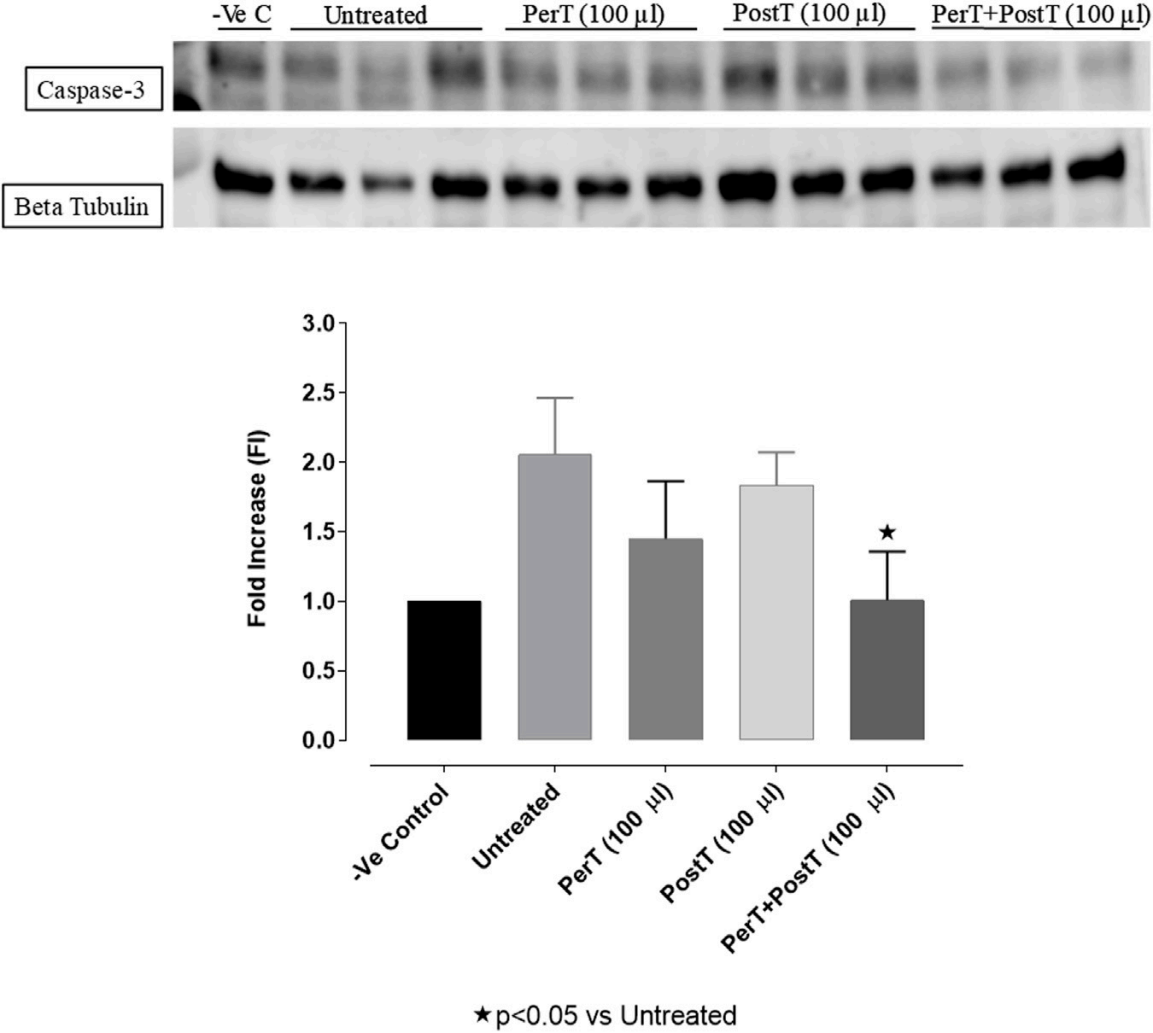
Caspase-3 activation at 10 min of reperfusion for experimental groups: Negative control (-Ve C), Untreated, BLS per-treatment (PerT), post-treatment (PostT) as well as BLS per- and post-treatment (PerT + PostT).

Even though not significant, BLS WIE marginally reduced Caspase-3 phosphorylation of BLS PerT (FI: 1.45 ± .24) and BLS PostT (FI: 1.83 ± 0.13), whereas Caspase-3 activation of the BLS PerT + PostT group (FI: 1.01 ± .20, *p* < 0.05) was significantly reduced vs. the untreated group (FI: 2.07 ± 0.23) (Figure 14).

#### PGC-1α

**FIGURE 15:**
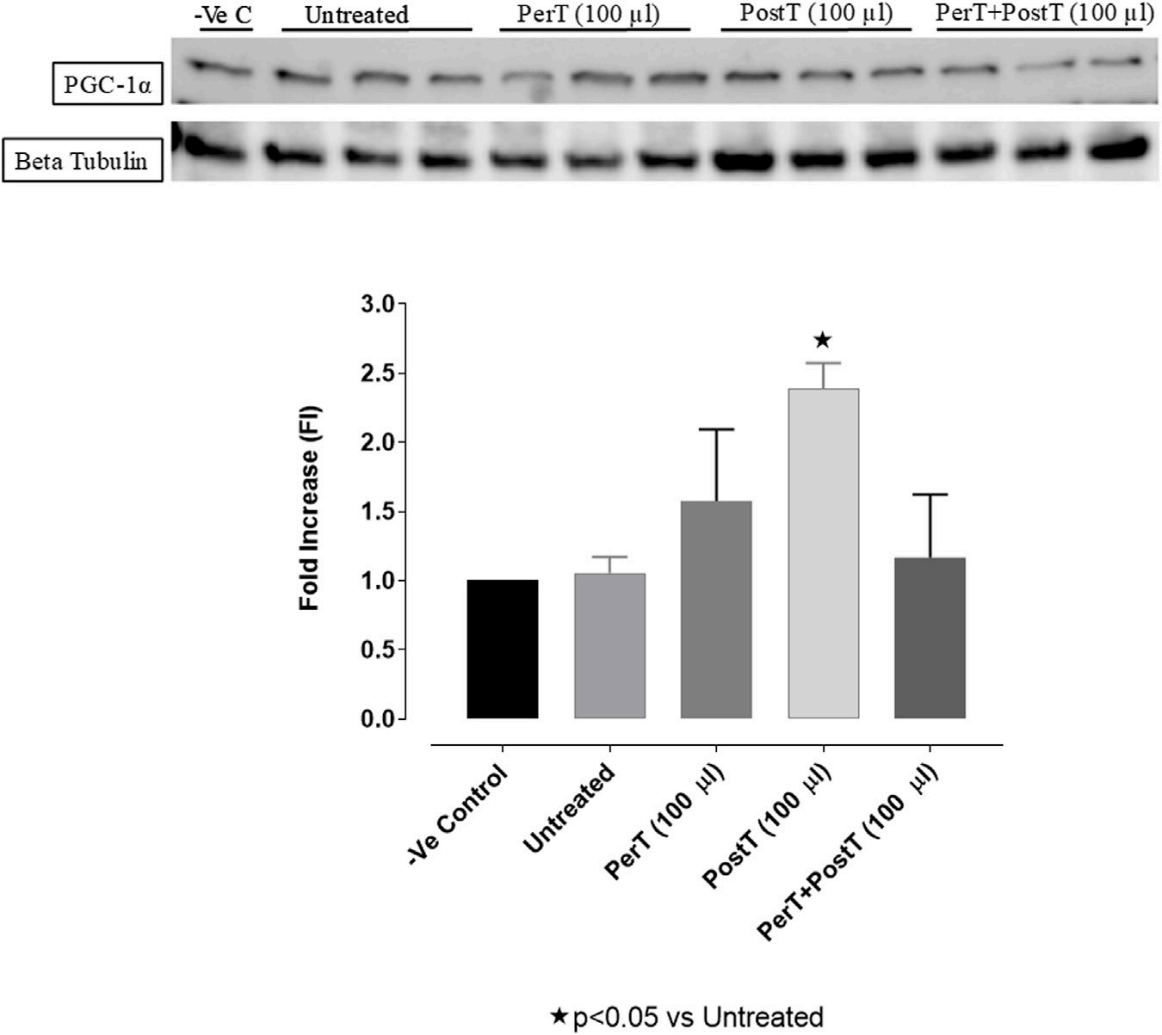
PGC1-α phosphorylation at 10 min reperfusion for experimental groups: Negative control (-Ve C), Untreated, BLS per-treatment (PerT), post-treatment (PostT), and BLS per- and post-treatment (PerT + PostT).

BLS PerT (FI: 1.67 ± 0.29) raised PGC-1α phosphorylation, even though not significant, whereas BLS PostT (FI: 2.93 ± 0.10) significantly increased PGC-1α phosphorylation when compared to the untreated group (FI: 1.07 ± 0.06, *p* < 0.05). BLS PerT + PostT (FI: 1.16 ± 0.26) had no effect on PGC-1α phosphorylation (Figure 15).

## Discussion

It is well recognized that diminished antioxidant mechanisms and ROS-induced oxidative stress has been linked with cardiovascular injury (Kilgore et al., 1999). However, low levels of ROS are crucial in physiological processes and feature prominently as key cardioprotective elements (Matsushima et al., 2014). Importantly, I/R contributes to ROS production and subsequent myocardial I/RI (Matsushima et al., 2013), which affects a plethora of cell signaling cascades, some of which may be cardioprotective. Notably, cardiovascular risk factors and comorbidities such as sex, age, hypertension, and metabolic diseases such as hyperlipidemia and diabetes (Ferdinandy et al., 2014), will ultimately impact the outcome of any treatment schedule. Consequently, it may be suitable to consider a multitarget cardioprotective therapy, such as BLS WIE which contain several polyphenolic compounds (Figures 2A–C). In this setting, BLS WIE may target multiple cardioprotective cascades, especially when applied at 3 different time periods of acute myocardial I/R; during ischemia as PerT, at reperfusion as PostT or late ischemia into early reperfusion as a PerT + PostT. Subsequently, the prospect of applying more than one cardioprotective therapy using one therapeutic agent at 3 phases of acute myocardial I/R may result in the activation and cohesion of multiple cardioprotective pathways.

### Ischaemia/reperfusion and the impact of BLS WIE on infarct size and functional recovery

The application of 100 µL BLS as BLS PerT; BLS PostT or as a BLS PerT + PostT, resulted in a significant infarct-sparring effect and improved functional recovery after I/R (Figures 4A, B).

### Myocardial ischaemia/reperfusion injury and the effect of BLS WIE on p-ERK p44/p42, p-Akt, Bax, Bcl, p-p38-MAPK, p-JNK, Nrf-2, NF-kB, Caspase-3, and PGC-1α activation

#### p-ERK p44/42, p-p38, and p-JNK

Moderate amounts of ROS (Redox signaling) are important elements in normal cellular processes, which may include activation of ERK, JNK as well as apoptosis signal-regulating kinase 1 (ASK-1), protein kinase C (PKC) and Protein kinase A (PKA) (Brennan et al., 2006). However, myocardial I/R injury induced oxidative stress has been linked with the activation of signal-regulating kinase 1 (AP-1), p-p38, p-JNK and the subsequent development of apoptosis and cell hypertrophy associated with cardiovascular injury (Hess and Manson, 1984). ROS also modulate cardioprotection in phenomena, such as ischaemic preconditioning (IPC) and beta preconditioning (BPC) (Baines et al., 1997). ERK activation as well reduce ROS production (Wang et al., 2021), creating a dynamic balance between activation of p-ERK, p-JNK and p-p38 that may determine whether a cell survives or succumb to apoptosis (Xia et al., 1995). In the milieu of crucial elements of BLS WIE, it should be noted that Rosmarinic acid (RA) is a key element detected in BLS WIE and significantly reduced TNF-α, IL-1β, IL 6, NO, p-JNK, p-p38 MAPK, and NF-kB when Rosmarinic acid was orally administered to streptozotocin (STZ)-induced type 2 Diabetes (T2D) Wistar rats (Govindaraj and Pillai, 2015). Other important polyphenolic diterpenes present in the BLS WIE is Carnosic acid, its derivative carnosol, which (Kocak et al., 2016) reduced myocardial damage, apoptosis, TNF-α levels, NF-kB, p38 MAPK, and pJNK 1/2 expressions as well as increased SOD activity, GSH-Px activity, p-ERK p44/p42, and Nrf-2 expressions.

In this model of I/R induced oxidative stress, only BLS PostT significantly increased p-ERK p44 activation. Even though BLS PerT and BLS PerT + PostT caused p-ERK p44 activation, this found not to be significant. A similar pattern was found with p-ERK p42 (Figure 5). However, this form of p-ERK p44/p42 activation was associated with significantly reduced IS (Figure 4A) at these specific time periods when BLS was applied as a PerT, PostT as well as BLS PerT + PostT. BLS PerT and BLS PostT had no effect onp-p38-MAPK or p-JNK p54 phosphorylation. However, p-p38-MAPK as well as p-JNK p46 phosphorylation was significantly decreased when the extract was applied as PerT at the end of ischaemia and continued as a PostT treatment at the onset of reperfusion (Figures 7, 8). These findings, highlight the effectiveness of BLS WIE when applied particularly at the end of ischaemia and continued as a PostT treatment at the onset of reperfusion, reducing I/R induced oxidative stress and curtailing cardiac damaging.

#### Nrf-2 and p-Akt

Nrf-2 controls transcriptional regulation of antioxidant enzymes and cytoprotective proteins, such as catalase (CAT), superoxide dismutase (SOD), heme oxygenase-1 (HO-1), and glutathione peroxidase (GPx) (Ruiz et al., 2013). Nrf-2 activity is enhanced by kinases, such as p-ERK, p-p38 (MAPK), PI3K and PKC, through the phosphorylation and inhibition of GSK-3β (Kaidanovich-Beilin and Woodgett, 2011). Notably, the application of BLS WIE during these critical time periods of I/R was shown to have beneficial effects, since BLS PerT, PostT as well as BLS PerT + PostT caused significant activation of Akt (Figure 6), which was associated with significantly increased Nrf-2 levels particularly at the onset of reperfusion with BLS PostT (Figure 13) and enhanced cardioprotective effects (Figure 3A), possibly due to a reduction of oxidative stress.

#### NF-kB

Recent evidence showed that NF-kB represses Nrf-2 signaling at transcription level (Li et al., 2008). In addition, Rosmarinic acid, a known inducer of Nrf-2, was also found to be cardioprotective against myocardial I/RI through suppression of the NF-kB inflammatory signaling pathway and ROS production in mice (Quana et al., 2021). Caffeic acid can reduce cardiac remodeling through downregulation of the MEK/ERK signaling pathway *in vivo* and *in vitro* (Ren et al., 2017). Caffeic acid also suppresses the NF-kB pathway by reduction of iNOS, COX-2 enzymes and the release of pro-inflammatory cytokines such as TNF-α, leading to the decrease of ROS production (Natarajan et al., 1996). Like Carnosic acid, Caffeic acid induces phosphorylation of Akt and AMP-activated protein kinase (AMPK), resulting in an increased GLUT4 expression, reduced pro-apoptotic factors and improving cell survival (Lee et al., 2015a). In the current study it was found that BLS PerT, BLS PostT or BLS PerT + PostT had no effect on NF-kB phosphorylation (Figure 12). This finding together with elevated Akt and Nrf-2 levels (Figure 13) as well as significantly reduced IS (Figure 4A), validating the inference of augmented antioxidant enzymes, the suppression of the NF-kB inflammatory signaling pathways and oxidative stress.

#### Bax, Bcl-2, and Caspase-3 activation

The Bcl-2 family of proteins, Bax and Bid are pro-apoptotic proteins, located mainly in the cytoplasm induces opening of the mitochondrial permeability transition pore (mPTP), whereas the anti-apoptotic proteins Bcl-2 and Bcl-xl, primarily located in the nuclear, mitochondrial and endoplasmic reticulum membranes. Opening of the mPTP subsequently, causes cytochrome-c (Cyt-c) release from the mitochondrial intermembrane into the cytoplasm (Yoo et al., 2019), where it binds to adenosine triphosphate, apoptotic protease-activating factor 1 (Apaf-1) and pro-caspase 9, which subsequently activates caspase-3 and results in DNA fragmentation (Pollack and Leeuwenburgh, 2001). A crucial role of RA in apoptosis was shown when RA pretreatment inhibited caspase-1 downstream signaling cascade, namely activation of caspase-3 and 9, release of Cyt-c and translocation of apoptosis-inducing factor (Jeong et al., 2011). It was also reported that carnosic acid enhanced the nuclear translocation of Nrf-2, upregulated the phase II/antioxidant enzyme activities as well as Bcl-2 levels and reduced myocardial expression of cleaved caspase-3, caspase-9, p53 and Bax (Sahu et al., 2014).

In this study, BLS WIE treatment, particularly during BLS PostT as well as BLS PerT + PostT, significantly minimized cellular damage of myocardial I/RI (Figure 4A). This cardioprotective response was shown to be triggered by activation of Akt (Figure 6) and increased Nrf-2 (Figure 13) as well as Bcl-2 levels (Figure 10). The activation of these cytoprotective proteins, unchanged Bax levels (Figure 9) and significantly reduced Caspase-3 levels (Figure 14), mediate increased antioxidant enzymes production, minimises oxidative stress and enhanced the cardioprotective effects of BLS WIE.

#### Bax/Bcl-2 ratio

Bcl-2 and Bcl-XL dimerize with pro-apoptotic proteins, such as Bax, to suppress apoptosis (Basu and Haldar, 1998). Since many cell types trigger Bcl-2 and Bax expression to suppress or enable apoptosis, the ratio of Bax/Bcl-2 has been considered as the independent regulator to determine apoptotic threshold (Harnois et al., 1997). Accordingly, cells with a high Bax/Bcl-2 ratio will be more sensitive to a given apoptotic stimulus when compared to a similar cell type with a comparatively low Bax/Bcl-2 ratio. It follows then, that the Bax/Bcl-2 ratio can be regarded as a valuable means to gauge of oxidative stress.

Salvigenin, a known polyphenol flavonoid, also shown to be present in BLS WIE, inhibits hydrogen peroxide-induced apoptosis, reduces the generation of ROS and reduces caspase-3 levels as well as Bax/Bcl-2 ratio in SH-Sy5Y neuroblastoma cells (Rafatian et al., 2012). In this study it was illustrated that BLS WIE effectively minimized precursors, such as p-caspase-3 levels as well as Bax/Bcl-2 ratio associated with ischaemia-reperfusion mediated oxidative stress. Subsequently, when comparing the relative Bax to the Bcl-2 expression levels (Bax/Bcl-2 ratio), it was found that the Bax/Bcl-2 ratio was significantly decreased with BLS (PerT), whereas the Bax/Bcl-2 ratio of BLS PostT as well as BLS (PerT + PostT) showed marginal but not significant decreases (Figure 11). Consequently, BLS WIE collectively reduced oxidative stress and provided effective cardioprotection.

#### PGC-1α

PGC-1 coactivates nuclear receptor transcription factors and Peroxisome proliferator-activated receptor gamma (PPAR) (Lehman et al., 2008), which regulate essential genes for mitochondrial fatty acids oxidation and cellular uptake. Importantly, high PGC-1α levels of have been found to be a critical protective factor in I/RI (Li et al., 2016) and in late cardiac ischemic preconditioning, PGC-1α is briefly induced during the transient ischaemic stress, which is accompanied with improved myocardial ischemia–reperfusion tolerance (Mcleod et al., 2004). In this study, PGC-1α phosphorylation was significantly increased when BLS WIE was administered, particularly at onset of reperfusion with BLS PostT (Figure 15). This was associated with significantly increased Nrf-2 levels (Figure 13), known to control transcriptional regulation of antioxidant enzymes and cytoprotective proteins, which is critical at the onset of reperfusion. However, continued high levels of PGC-1α may result in adverse myocardial function and stress susceptibility. Overexpression of PGC-1α can result in a progressive and uncontrolled surge in mitochondrial number, disruption in cardiomyocyte sarcomeric structure and myocardial contractile function (Lehman et al., 2000). Although BLS PerT + PostT was associated with lowered PGC-1α levels, cardioprotection was still maintained, as illustrated by the reduced IS. It can be ventured to say that higher PGC-1α levels was not necessary with BLS PerT + PostT, since PGC-1α phosphorylation prior to ischeamie (BLS PerT) was adequate to ensure cardioprotection at the onset of reperfusion.

## Summary

**FIGURE 16:**
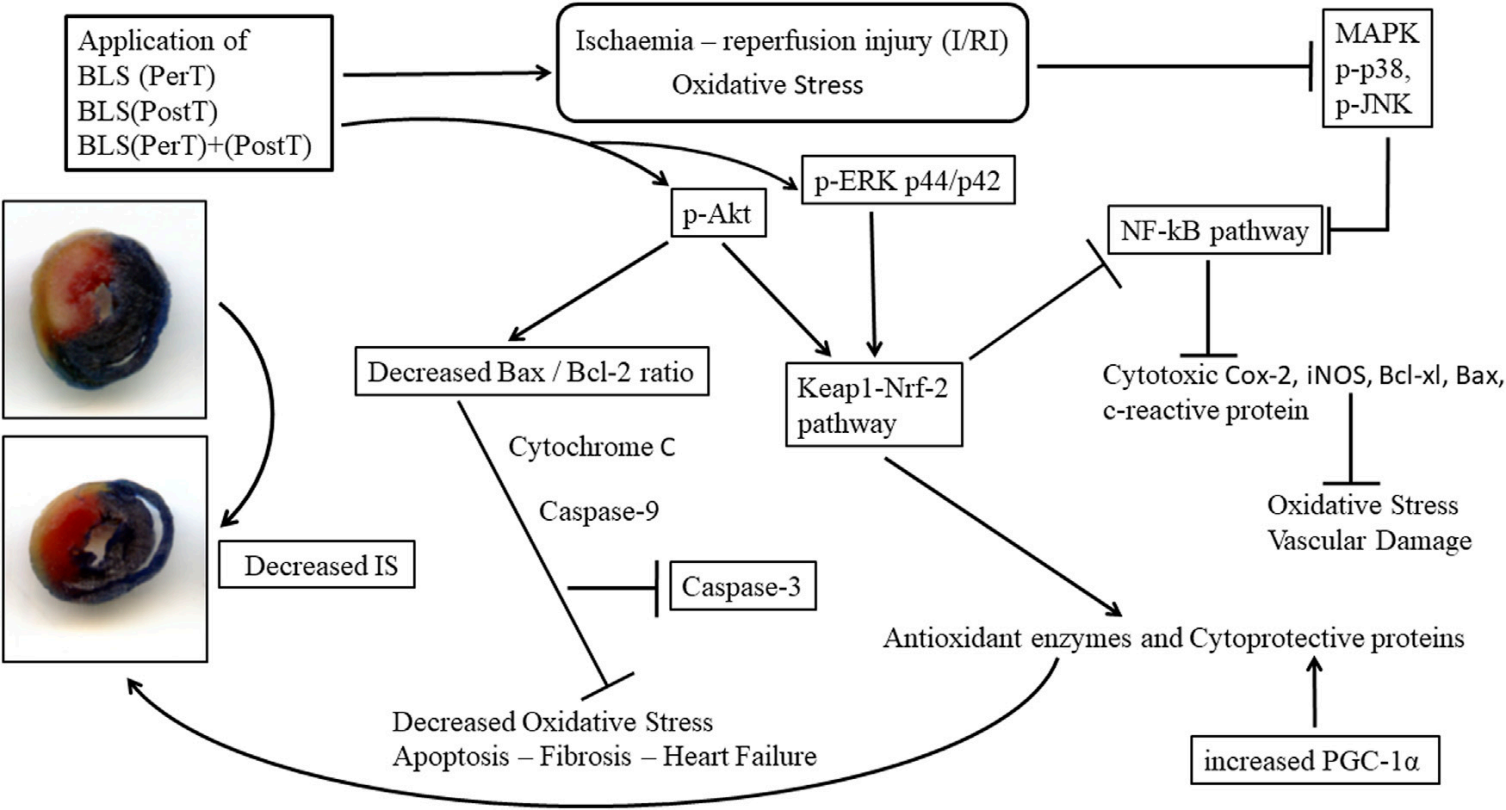
Schematic representation of the administration of polyphenol-rich BLS WIE at different stages of ischaemia and/or reperfusion; activate/inhibit several signaling events simultaneously and mediate cardioprotection in a multitarget manner.

The regulation of ROS signaling pathways are essential to prevent ROS overproduction associated with I/R induced oxidative stress and cardiac damage. In this model of I/R induced oxidative stress, only BLS PostT significantly increased p-ERK p44 activation. Even though BLS PerT and BLS PerT + PostT triggered high levels of p-ERK p44/p42 activation, this was found not to be significant (Figure 5). However, IS was still significantly reduced (Figure 4A) at these specific time periods when BLS was applied as a PerT, PostT as well as BLS PerT + PostT. p-p38-MAPK (Figure 7) as well as p-JNK p46 phosphorylation (Figure 8) was significantly decreased when the extract was applied as a PerT at the end of ischaemia and continued as a PostT treatment at the onset of reperfusion. The cardioprotective response of BLS PostT as well as BLS PerT + PostT (Figure 4A) was also associated with significantly increased p-Akt (Figure 6), Nrf-2 (Figure 13) as well as Bcl-2 levels (Figure 10). The suppression of NF-kB inflammatory signaling pathways (Figure 12) at these time periods, validate the augmentation of antioxidant enzyme activity and enhanced cardioprotection. The activation of these cytoprotective proteins, unchanged Bax levels (Figure 9) and significantly decreased Bax/Bcl-2 ratios with BLS (PerT), marginal but not significantly decreased Bax/Bcl-2 ratios of PostT as well as BLS (PerT + PostT) (Figure 11), illustrates the capabilities of BLS WIE to reduce oxidative stress and bring about effective cardioprotection. This was substantiated by significantly reduced Caspase-3 levels (Figure 14) and significantly increased PGC-1α phosphorylation when BLS WIE was administered particularly at onset of reperfusion with BLS PostT (Figure 15). This finding was associated with significantly increased Nrf-2 levels (Figure 13), known to control transcriptional regulation of antioxidant enzymes, cytoprotective proteins and enhanced cardioprotection. The application of BLS WIE which contain several polyphenolic, cardioprotective compounds administered at different stages of the ischaemia and/or reperfusion, simultaneously targeted several signaling events associated with I/RI to achieve a better cardioprotective outcome in a multitarget cardioprotective manner (Figure 16).

## Figures and Tables

**Table:** 

**Apaf-1-**	Apoptotic protease-activating factor 1
**AR-**	Area at risk
**ASK-1**	Apoptosis signal-regulating kinase 1
**Bax**	Bcl-2-associated X protein
**Bcl-2**	B-cell lymphoma 2
**BLS**	Blue Sage
**BPC**	Beta preconditioning
**Caspase-3**	Cysteine-dependent aspartate-directed proteases
**CAT**	Catalase
**CO**	cardiac output
**Cyt-c**	Cytochrome-c
**ECRA**	Ethics Committee for Research on Animals
**ERK p44/p42**	Extracellular signal-regulated kinases p44/p42
**GPx**	Glutathione peroxidase
**HO-1**	Heme oxygenase-1
**I**	Area of infarct tissue
**I/R**	ischaemia—reperfusion
**I/RI**	ischaemia—reperfusion injury
**IPC**	Ischaemic preconditioning
**IS**	Infarct size
**JNK**	Jun N-terminal kinase
**LC-MS**	Liquid chromatography–mass spectrometry
**NF-kB**	Nuclear factor kappa B
**Nrf-2**	Nuclear factor erythroid 2–related factor 2
**p38-MAPK**	p38 mitogen-activated protein kinases
**p-Akt**	p-Ak strain transforming
**% FR**	Percentage functional recovery
**% I/AR**	Percentage of the area at risk
**PerT**	Per-treatment
**PerT + PostT**	Per- and Post-treatment
**PGC-1α**	Peroxisome proliferator activated receptor gamma coactivator-1 alpha
**PostT**	Post-treatment
**PPAR**	Peroxisome proliferator-activated receptor gamma
**Qa**	Aortic flow rate
**Qc**	Coronary flow rate
**RA**	Rosmarinic acid
**RI**	Regional ischaemia (RI)
**ROS**	Reactive oxygen species
**SOD**	Superoxide dismutase
**UT**	Untreated group
**-Ve C**	Negative control group
**WHO**	World Health Organization
**WIE**	Water infusion extract

## Data Availability

The raw data supporting the conclusion of this article will be made available by the authors, without undue reservation.
